# Decoupling Ultralow Coherent and Particle‐Like Phonon Transport via Bonding Hierarchy in Soft Superionic Crystals

**DOI:** 10.1002/advs.202506807

**Published:** 2025-06-05

**Authors:** Wenjie Xiong, Hao Huang, Yu Wu, Xinji Xu, Geng Li, Zonglin Gu, Shuming Zeng

**Affiliations:** ^1^ College of Physics Science and Technology Yangzhou University Jiangsu 225009 China; ^2^ Advanced Copper Industry College Jiangxi University of Science and Technology Yingtan 335000 China; ^3^ Advanced Thermal Management Technology and Functional Materials Laboratory, Ministry of Education Key Laboratory of NSLSCS, School of Energy and Mechanical Engineering Nanjing Normal University, Jiangsu Nanjing 210023 China; ^4^ China Rare Earth Group Research Institute Shenzhen Guangdong 518000 China; ^5^ Key Laboratory of Rare Earths, Ganjiang Innovation Academy Chinese Academy of Sciences Ganzhou 341000 China

**Keywords:** bonding hierarchy, lattice thermal conductivity lower limit, phonon flat band, soft superionic crystals

## Abstract

Within the unified theoretical framework for thermal transport, the inherent interplay between coherent tunneling and propagative phonon mechanisms establishes an antagonistic relationship, thereby imposing fundamental limitations on suppressing lattice thermal conductivity $\kappa_{L}$. In this work, it is theoretically demonstrate that the superionic crystals X_6_Re_6_S_8_I_8_ (X = Rb, Cs) exhibit ultralow glass‐like and particle‐like thermal conductivities. The weak interactions between free alkali metal ions X^+^ (X = Rb, Cs) and I^−^ anions induce pronounced lattice anharmonicity, which enhances phonon scattering and suppresses group velocities, thereby reducing the particle‐like thermal conductivity ($\kappa_{p}$). Concurrently, the significant bonding heterogeneity within and between the [Re_6_S_8_I_6_]^4 −^ clusters promotes phonon band flattening and low‐frequency phonon localization. The resulting discretized phonon flat bands substantially diminish the glass‐like thermal conductivity ($\kappa_{c}$). At room temperature, the total $\kappa_{L}$ of X_6_Re_6_S_8_I_8_ (X = Rb, Cs) falls below 0.2 W m^−1^ K^−1^. Furthermore, the bonding characteristics between X^+^ and I^−^ anions induce an anomalous cation mass‐independent stiffening of low‐frequency phonon branches in this system, resulting in counterintuitive thermal transport behavior. This work elucidates fundamental mechanisms governing heat transfer in ultralow $\kappa_{L}$ materials and establishes novel pathways for transcending conventional thermal conductivity limitations.

## Graphical abstract

The bonding hierarchy in X_6_Re_6_S_8_I_8_ (X = Rb, Cs) promotes phonon localization, generating extensive discrete phonon flat bands that redefine the lower bounds of lattice thermal conductivity in crystals.
 

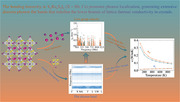

## 1 Introduction

The exploration of materials with ultralow lattice thermal conductivity $\kappa_{L}$ holds significant importance in both fundamental research and technological applications, such as thermal barrier coatings^[^ ^1^ ^]^ and thermoelectrics.^[^ ^2^ ^]^ Materials with intrinsically low $\kappa_{L}$, typically governed by phonon–phonon scattering mechanisms, exhibit several distinctive features such as intrinsic rattling,^[^ ^3^ ^]^ lattice anharmonicity,^[^ ^4^ ^]^ ferroelectric instability,^[^ ^5^ ^]^ and structural complexity.^[^ ^6^ ^]^ Based on these principles, a series of materials with low $\kappa_{L}$ at room temperature such as Tl_3_VSe_4_ (0.30 W m^−1^ K^−1^),^[^ ^7^ ^]^ TlSe (0.62 W m^−1^ K^−1^),^[^ ^8^ ^]^ and Cu_12_Sb_4_S_13_ (0.67 W m^−1^ K^−1^)^[^ ^6^ ^]^ have been discovered. In addition, strategies like nanostructuring and entropy‐driven point defect engineering can further suppress $\kappa_{L}$.^[^ ^9^ ^]^ The advent of machine learning methods has enabled efficient exploration of low $\kappa_{L}$ materials across vast chemical and structural spaces.^[^ ^10^ ^]^ However, the $\kappa_{L}$ of crystalline materials cannot be arbitrarily low‐it is fundamentally bounded.^[^ ^11^ ^]^ To date, the experimentally measured $\kappa_{L}$ of crystals at room temperature has not fallen below 0.2 W m^−1^ K^−1^, the search for materials with ultralow $\kappa_{L}$ remains an active research frontier.

In the unified theory of lattice thermal conductivity proposed by Simoncelli et al.,^[^ ^12^, ^13^ ^]^ heat transport is governed by dual contributions from localized diffusons (glass‐like thermal conductiviy $\kappa_{c}$) and propagating phonons (particle‐like thermal conductivity $\kappa_{p}$). Generally, in simple lattices with few atoms per primitive cell (PC), $\kappa_{L}$ is dominated by propagating phonons. However, in complex crystals with many atoms per PC, the contribution of localized diffusons becomes significant and cannot be neglected. For example, at 300 K, PbTe (two atoms per PC) exhibits a total $\kappa_{L}$ of 2 W m^−1^ K^−1^, which is almost entirely contributed by $\kappa_{p}$.^[^ ^14^ ^]^ In contrast, the complex crystal Ag_8_GeS_6_ (60 atoms per PC) demonstrates an ultralow $\kappa_{p}$ of merely 0.04 W m^−1^ K^−1^, which comparable to that of air, while its $\kappa_{c}$ reaches 0.43 W m^−1^ K^−1^, significantly exceeding $\kappa_{p}$.^[^ ^11^ ^]^ A viable strategy for discovering materials with ultralow $\kappa_{L}$ involves either minimizing $\kappa_{p}$ in simple lattices or suppressing $\kappa_{c}$ in complex crystal structures. For the former case, Zeng et al. identified AgTlI_2_ (I4/mcm) as a rare simple crystalline system simultaneously exhibiting low $\kappa_{p}$ and suppressed $\kappa_{c}$,^[^ ^11^ ^]^ achieving an unprecedented room‐temperature $\kappa_{L}$ of 0.25 W m^−1^ K^−1^. For the latter case (complex crystals), whether materials with intrinsically low $\kappa_{c}$ exist remains an open fundamental question in thermal transport physics.

In this work, we systematically investigate the lattice thermal conductivity of the recently discovered superionic compounds^[^ ^15^, ^16^, ^17^ ^]^ X_6_Re_6_S_8_I_8_ (X = Rb, Cs)^[^ ^18^ ^]^ using first‐principles calculations combined with the temperature‐dependent effective potential (TDEP) method. By rigorously treating three‐ and four‐phonon scattering processes within the unified theory of lattice thermal transport, we reveal exceptionally suppressed contributions from both $\kappa_{p}$ and $\kappa_{c}$, leading to an ultralow $\kappa_{L}$ below 0.2 W m^−1^ K^−1^. The quasi 0D structure of X_6_Re_6_S_8_I_8_ consists of isolated [Re_6_S_8_I_6_]^4 −^ clusters embedded in a 3D ionic framework formed by Rb^+^/Cs^+^ cations and bridging I^−^ anions. Strong phonon anharmonicity and localization in this architecture generate extensive optical flat bands that suppress both particle‐like and wave‐like thermal transport. Counterintuitively, despite the heavier atomic mass of Cs‐which typically reduces phonon group velocities (*v*_g_) ‐our calculations reveal higher thermal conductivity in Cs‐based compounds than in their Rb analogs. This anomaly arises from distinct X‐I interactions that reshape local lattice dynamics and bonding, unexpectedly enhancing phonon lifetimes and propagation in Cs variants. These results demonstrate that even materials with complex structures can exhibit negligible $\kappa_{c}$, establishing new principles for engineering ultralow $\kappa_{L}$ materials.

## 2 Computational Methods

All first‐principles calculations were performed using the Vienna Ab Initio Simulation Package,^[^ ^19^ ^]^ based on density functional theory.^[^ ^20^, ^21^ ^]^ The projector augmented wave method^[^ ^22^ ^]^ with a plane‐wave basis set was employed to describe ion‐electron interactions, while exchange‐correlation effects were treated within the revised Perdew‐Burke‐Ernzerhof functional optimized for solids.^[^ ^23^ ^]^ A plane‐wave energy cutoff of 520 eV was imposed throughout the calculations. Structural relaxations utilized a 6 × 6 × 6 Monkhorst‐Pack **k**‐mesh for Brillouin zone sampling, with convergence thresholds of 10^−8^ eV for energy and 10^−4^ eVÅ^−1^ for atomic forces. Born effective charge tensors and dielectric constants were computed via density functional perturbation theory,^[^ ^24^ ^]^ incorporating nonanalytic corrections to the dynamical matrix. To determine interatomic force constants (IFCs), we conducted ab initio molecular dynamics (AIMD) simulations in the NVT ensemble using a 2× 2 ×2 supercell containing 224 atoms. The system was equilibrated for 20 ps with a timestep of 1 fs. Temperature‐dependent effective potential methodology^[^ ^25^ ^]^ was subsequently applied to extract second‐, third‐, and fourth‐order IFCs at varying temperatures, employing cutoff radii of 8, 6, and 4 Å respectively. These cutoff values ensured comprehensive inclusion of atomic interactions governing the material's dynamical response.

Within the Wigner formalism, the lattice thermal conductivity tensor $\kappa_{L}^{\alpha \beta }$ decomposes into two contributions:

(1) $$\kappa_{L}^{\alpha \beta }=\kappa_{p}^{\alpha \beta }+\kappa_{c}^{\alpha \beta }$$

where the particle‐like term $\kappa_{p}^{\alpha \beta }$ describes semiclassical phonon transport:

(2) $$\kappa_{p}^{\alpha \beta }=\frac{1}{N_{q}V}\sum_{\mathrm{qs}}C_{q}^{s}\nu_{q,\alpha }^{s}\nu_{q,\beta }^{s}\tau_{q}^{s}$$

here, $C_{q}^{s}=\hbar \omega_{q}^{s}\partial n_{q}^{s}/\partial T$ denotes the mode‐resolved heat capacity, with $n_{q}^{s}$ being the Bose–Einstein distribution. The coherence contribution $\kappa_{c}^{\alpha \beta }$ captures wave‐like tunneling effects between distinct phonon branches *s* and *s*′:

(3) $$\begin{array}{ccc} \kappa_{c}^{\alpha \beta } & = & \frac{\hbar^{2}}{k_{B}T^{2}VN_{q}}\sum_{q}\sum_{s\neq s^{\prime }}\frac{\omega_{q}^{s}+\omega_{q}^{s^{\prime }}}{2}\nu_{q,\alpha }^{s,s^{\prime }}\nu_{q,\beta }^{s,s^{\prime }}\\ & & \times \frac{\omega_{q}^{s}n_{q}^{s}\left(n_{q}^{s}+1\right)+\omega_{q}^{s^{\prime }}n_{q}^{s^{\prime }}\left(n_{q}^{s^{\prime }}+1\right)}{4{\left(\omega_{q}^{s}-\omega_{q}^{s^{\prime }}\right)}^{2}+{\left(\Gamma_{q}^{s}+\Gamma_{q}^{s^{\prime }}\right)}^{2}}\left(\Gamma_{q}^{s}+\Gamma_{q}^{s^{\prime }}\right) \end{array}$$

where $\nu_{q,\alpha }^{s,s^{\prime }}$ represents the interband velocity matrix element. The Cartesian indices α, β ∈ {*x*, *y*, *z*} span spatial dimensions, *V* is the unit cell volume, and *N*_*q*_ enumerates sampled phonon wavevectors in the Brillouin zone. Scattering rates $\Gamma_{q}^{s}$ quantify phonon lifetime broadening. This dual formulation reconciles semiclassical transport ($\kappa_{p}$) with quantum coherence effects ($\kappa_{c}$), particularly significant in materials exhibiting phonon branch degeneracies or strong anharmonicity. The $\kappa_{L}^{\alpha \beta }$ was computed using an in‐house modified version of the FourPhonon code,^[^ ^26^, ^27^, ^28^, ^29^ ^]^ incorporating three‐ and four‐phonon scattering processes, and performed in a *q*‐mesh of 10 × 10 × 10. Figure S1 (Supporting Information) shows the calculated lattice thermal conductivity at different *q*‐points. It can be concluded that the $\kappa_{L}$ values converge when the *q*‐point density is greater than 8 × 8 × 8.

## 3 Results and Discussion

**Figure 1:**
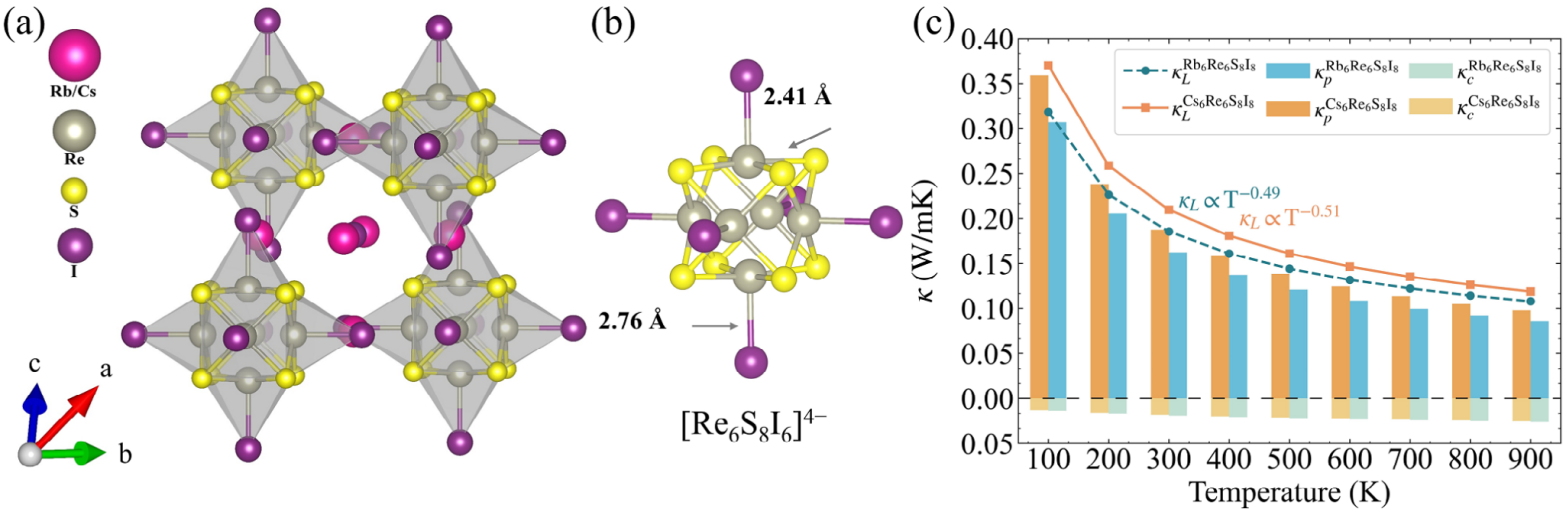
a)The crystal structure of X_6_Re_6_S_8_I_8_ (X = Rb, Cs); b) The Isolated [Re_6_S_8_I_6_]^4−^ cluster; c) The total lattice thermal conductivities ($\kappa_{L}$), the particle‐like lattice thermal conductivities ($\kappa_{p}$) and the glass‐like lattice thermal conductivities ($\kappa_{c}$) of X_6_Re_6_S_8_I_8_ (X = Rb, Cs) from 100 to 900K.

The superionic crystals X_6_Re_6_S_8_I_8_ (X = Rb, Cs) adopt a cubic 0D perovskite structure,^[^ ^30^ ^]^ with Rb_6_Re_6_S_8_I_8_ having been experimentally synthesized.^[^ ^18^ ^]^ As shown in **Figure** **1**a, X_6_Re_6_S_8_I_8_ crystallizes in the space group $Fm\bar{3}m$, comprising alkali metal ions (X^+^), iodide ions (I^−^), and discrete [Re_6_S_8_I_6_]^4 −^ clusters. The [Re_6_S_8_I_6_]^4 −^ cluster (Figure 1b) serves as the fundamental building unit, featuring an octahedral Re_6_ core stabilized by direct metal–metal bonds (Re‐Re: 2.60  Å). Each face of the Re_6_ octahedron is capped by a sulfur atom in µ_3_ coordination (Re‐S: 2.41  Å), forming a [Re_6_S_8_]^2 +^ core. Peripheral iodides coordinate to the octahedron through Re‐I bonds (2.76  Å), completing the [Re_6_S_8_I_6_]^4−^ cluster. Alkali metal atoms (X) occupy interstitial sites between clusters, exhibiting weak X‐I interactions (Rb‐I: 3.94  Å) that stabilize the crystal framework. Additional X‐I‐X bridging motifs (Rb‐I: 3.88  Å) fill structural voids, forming ionic coordination networks. Optimized structural parameters for Rb_6_Re_6_S_8_I_8_ show agreement with experimental data.^[^ ^18^ ^]^ Detailed structural parameters for both Rb and Cs analogs are provided in Table SI (Supporting Information). The system exemplifies a rattling model, where X atoms (Rb/Cs) behave as loosely bound“rattler” guests within the rigid [Re_6_S_8_I_6_]^4−^ host framework.

The electronic structures of X_6_Re_6_S_8_I_8_ are presented in Figure S2 (Supporting Information). Both Rb_6_Re_6_S_8_I_8_ and Cs_6_Re_6_S_8_I_8_ exhibit indirect bandgap semiconductor behavior, with valence band maxima (VBM) at the Γ‐point and conduction band minima (CBM) at the *L*‐point, yielding bandgaps (*E*_*g*_) of 2.42 and 2.45 eV, respectively. Atomic projected electronic density of states (EDOS) analysis reveals dominant contributions from S and I atomic orbitals near the VBM, while Re atomic orbitals primarily constitute the CBM. To assess thermal stability, we performed 20 ps NVT‐ensemble AIMD simulations at varying temperatures (see Figure S3, Supporting Information). Remarkably, both compounds maintain structural integrity up to 800 K, evidenced by minimal fluctuations in total energy. Phonon dispersion calculations within the harmonic approximation confirm dynamical stability, showing no imaginary frequencies across the Brillouin zone (Figure S4, Supporting Information). This stability persists under finite‐temperature conditions as validated by TDEP calculations up to 800 K. Notably, temperature evolution induces distinct phonon renormalization: mid‐to‐high‐frequency optical branches soften with increasing temperature, whereas low‐frequency optical modes exhibit hardening behavior. This anomalous temperature dependence correlates with atomic vibration patterns – mid/high‐frequency modes predominantly involve Re‐S framework vibrations, while low‐frequency modes arise from I‐Rb/I‐Cs interactions, as shown in Figure S6 (Supporting Information). The hardening of low‐frequency optical branches suggests enhanced anharmonic coupling between Rb^+^ and I^−^ ionic bonds at elevated temperatures.

The cage‐like crystal structure of X_6_Re_6_S_8_I_8_ (X = Rb, Cs) engenders distinctive thermal transport characteristics.^[^ ^31^ ^]^ Figure 1c presents the temperature‐dependent $\kappa_{L}$ ($\kappa_{L}$ = $\kappa_{p}$ + $\kappa_{c}$) decomposition, revealing particle‐like $\kappa_{p}$ and coherent $\kappa_{c}$ contributions. At 300 K, Cs_6_Re_6_S_8_I_8_ and Rb_6_Re_6_S_8_I_8_ exhibit ultralow κ_L_ values of 0.19 and 0.17 W m^−1^ K^−1^, respectively, surpassing recently reported ultralow κ_L_ materials including Ag_8_GeTe_6_ and AgTlI_2_, as well as conventional thermoelectric materials (see **Table** **2**). Notably, both compounds demonstrate weak temperature dependence with $\kappa_{L}$ ∝*T* ^−0.51^ (Cs) and *T* ^−0.49^ (Rb), signaling strong lattice anharmonicity. The particle contribution dominates the thermal transport, accounting for 90.2% (Cs) and 87.9% (Rb) of $\kappa_{L}$ at 300 K. This hierarchy suggests that diffusive phonon transport governs heat conduction in these superionic crystals. Intriguingly, the heavier Cs analog exhibits higher $\kappa_{L}$ than its Rb counterpart – a mass dependence reversal contradicting conventional Slack formalism predictions.^[^ ^32^, ^33^ ^]^ Structural analysis (Table S1, Supporting Information) reveals significant lattice parameter and bond length variations between the analogs. These structural distinctions likely modify interatomic force constants, thereby altering phonon scattering phase space.

**Table 1:** The elastic constants (C_*ij*_), the bulk modulus (B), shear modulus (G), Young's modulus (Y), and average wave velocity (υ) of X_6_Re_6_S_8_I_8_, unit in GPa.

Material	*C*_11_	*C*_12_	*C*_44_	*B*	*G*	*E*
Rb_6_Re_6_S_8_I_8_	14.54	7.75	5.36	10.01	4.58	11.91
Cs_6_Re_6_S_8_I_8_	16.69	5.55	6.11	9.27	5.90	14.59

**Table 2:** Comparative sound velocities (υ) and lattice thermal conductivities at 300 K.

Material	Sound velocity υ [m s^−1^]	κ_*L*_ [W m^−1^K^−1^]
**Rb_6_Re_6_S_8_I_8_** (this work)	1040.78	0.17
Ag_8_GeTe_6_	1099	0.25
**Cs_6_Re_6_S_8_I_8_** (this work)	1163.06	0.19
Ag_8_SnSe_6_ ^†^	1494	0.36
Cu_8_GeSe_6_ ^†^	1793	0.3
PbSe†	1805	1.5
PbTe†	1814	1.9

^†^ Values from references [34, 35].

For cubic symmetry systems, the independent elastic tensor components reduce to *C*_11_, *C*_12_, and *C*_44_, as presented in **Table** **1**. The elastic anisotropy factor (*A*), characterizing mechanical property variations along different crystallographic directions, is defined as: $A=\frac{2C_{44}}{C_{11}-C_{12}}$. Our calculations yield *A* = 1.58 for Rb_6_Re_6_S_8_I_8_ and *A* = 1.10 for Cs_6_Re_6_S_8_I_8_, both approaching unity, indicative of quasi‐isotropic mechanical behavior. Notably, the larger anisotropy in Rb_6_Re_6_S_8_I_8_ suggests directional constraints in phonon propagation that may enhance scattering processes. Comparative analysis of elastic moduli reveals weaker interatomic bonding and a more loosely packed structure in Rb_6_Re_6_S_8_I_8_, as evidenced by its lower elastic modulus. This structural characteristic correlates with enhanced lattice anharmonicity, which restricts phonon transport velocities. Supporting this interpretation, Table 2 demonstrates substantially reduced sound velocities in Rb_6_Re_6_S_8_I_8_ compared to its cesium counterpart. Significantly, the X_6_Re_6_S_8_I_8_ system exhibits a remarkably low $\kappa_{L}$ comparable to traditional thermoelectric materials, suggesting promising potential for energy conversion applications.

**Figure 2:**
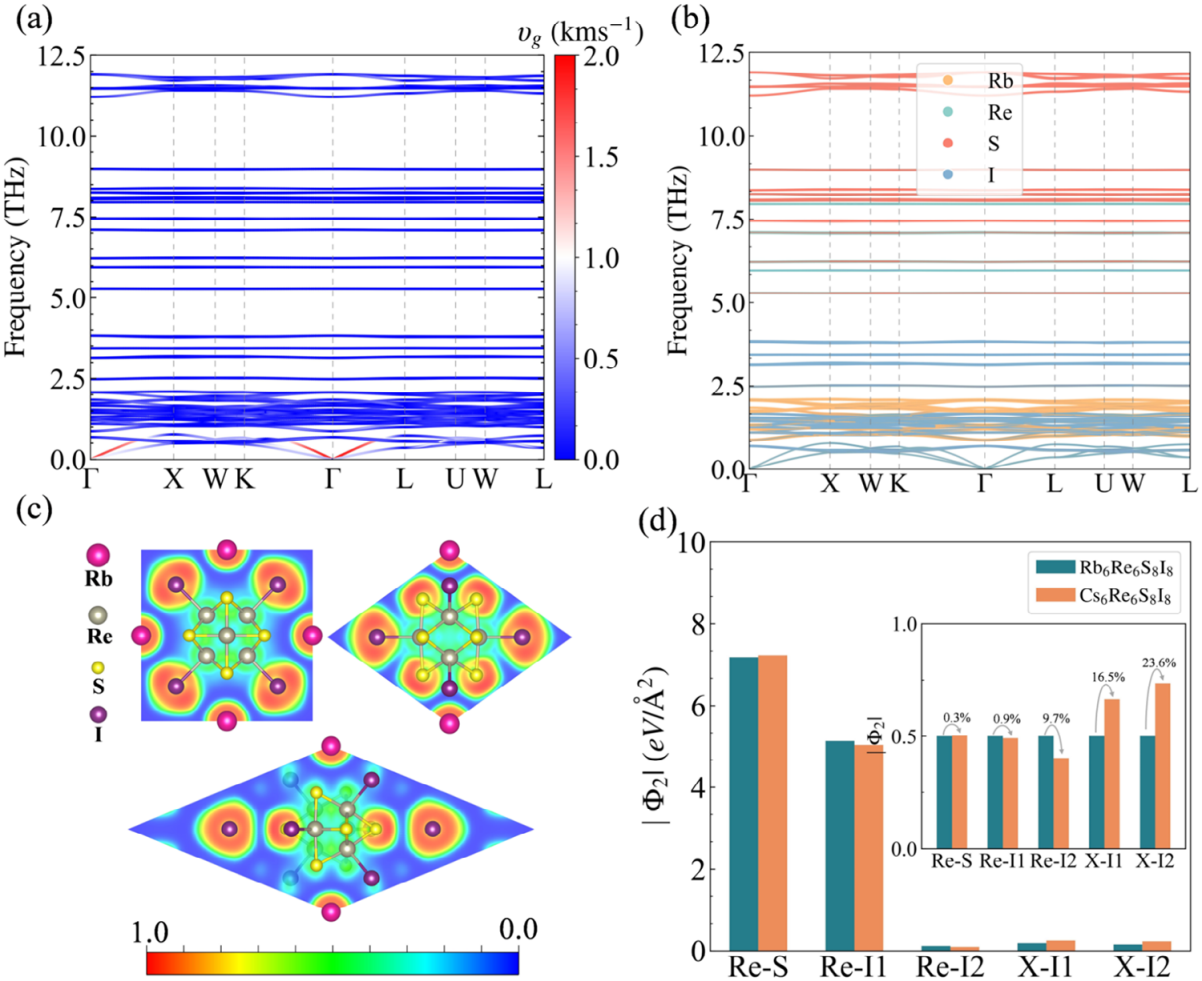
a)The phonon dispersion of Rb_6_Re_6_S_8_I_8_ at 300 K with group velocities *v*_g_ projection; b) The projected phonon dispersion with atomic projection of Rb_6_Re_6_S_8_I_8_ at 300 K; c) The ELF corresponding to the (0,1,1), (1,2,1), and (‐1,2,‐1) planes of Rb_6_Re_6_S_8_I_8_; d) The norm of the second‐order force constants |Φ_2_| for the top three strongest bonds in X_6_Re_6_S_8_I_8_ (X = Rb, Cs). The center is the results of the relative value of the norm of the |Φ_2_| in X_6_Re_6_S_8_I_8_ (X = Rb, Cs).

To elucidate the origin of the low lattice thermal conductivity κ_L_ in X_6_Re_6_S_8_I_8_ compounds, we take Rb_6_Re_6_S_8_I_8_ as a prototype system. **Figure** **2**a displays the projected phonon group velocities *v*_g_ along the phonon dispersion at 300 K. The phonon dispersion exhibits remarkable flatness across the entire frequency spectrum, particularly in optical branches, indicating spatially localized vibrational modes that hinder efficient energy propagation. All phonon modes demonstrate exceptionally low *v*_g_, with maximum values below 2 km s^−1^ and majority below 0.5 km s^−1^, signifying sluggish heat transfer kinetics. The projected phonon eigenvectors in Figure 2b reveal distinct vibrational characteristics: acoustic branches primarily involve I atoms, while low‐frequency optical branches (<2.5 THz) arise from coupled vibrations of Rb and I atoms. Notably, these low‐frequency modes (<2.5 THz) dominate thermal transport, suggesting significant contributions from Rb–I vibrations to κ_L_. Both phonon dispersion and group velocity characteristics correlate strongly with interatomic bonding environments. Figure 2c and Figure S5 (Supporting Information) present the electron localization function (ELF) analysis, complemented by Bader charge calculations in **Table** **3**. Clearly, two distinct iodine species emerge: 1) Covalent I1 within the [Re_6_S_8_I_6_]^4 −^ octahedral clusters shows moderate electron localization (ELF ≈ 0.5) with incomplete charge transfer from Re, and 2) Ionic I exhibits negligible electron localization (ELF ≈ 0). The [Re_6_S_8_I_6_]^4−^ clusters maintain partial covalent bonding (Re–S/I ELF ≈ 0.5), while Rb atoms demonstrate complete electron transfer (ELF ≈ 0) characteristic of ionic bonding. This dual bonding architecture – stable covalent clusters interspersed with ionic Rb^+^ and I^−^ species – creates weakly bonded structural frameworks. The resulting soft inter‐cluster interactions promote extensive phonon band flattening, particularly in optical branches, ultimately suppressing κ_L_ through enhanced phonon scattering and reduced phonon group velocities.

**Table 3:** Bader effective charges of constituent atoms in X_6_Re_6_S_8_I_8_ (X = Rb, Cs), unit in e^−^.

Element	Formal charge	Bader effective charge	
		X = Rb	X = Cs
Rb/Cs	+1	0.836	0.999
Re	+3	0.784	0.603
S	−2	−0.668	−0.648
I_1_	−1	−0.480	−0.456
I_2_	−1	−0.751	−0.850

**Figure 3:**
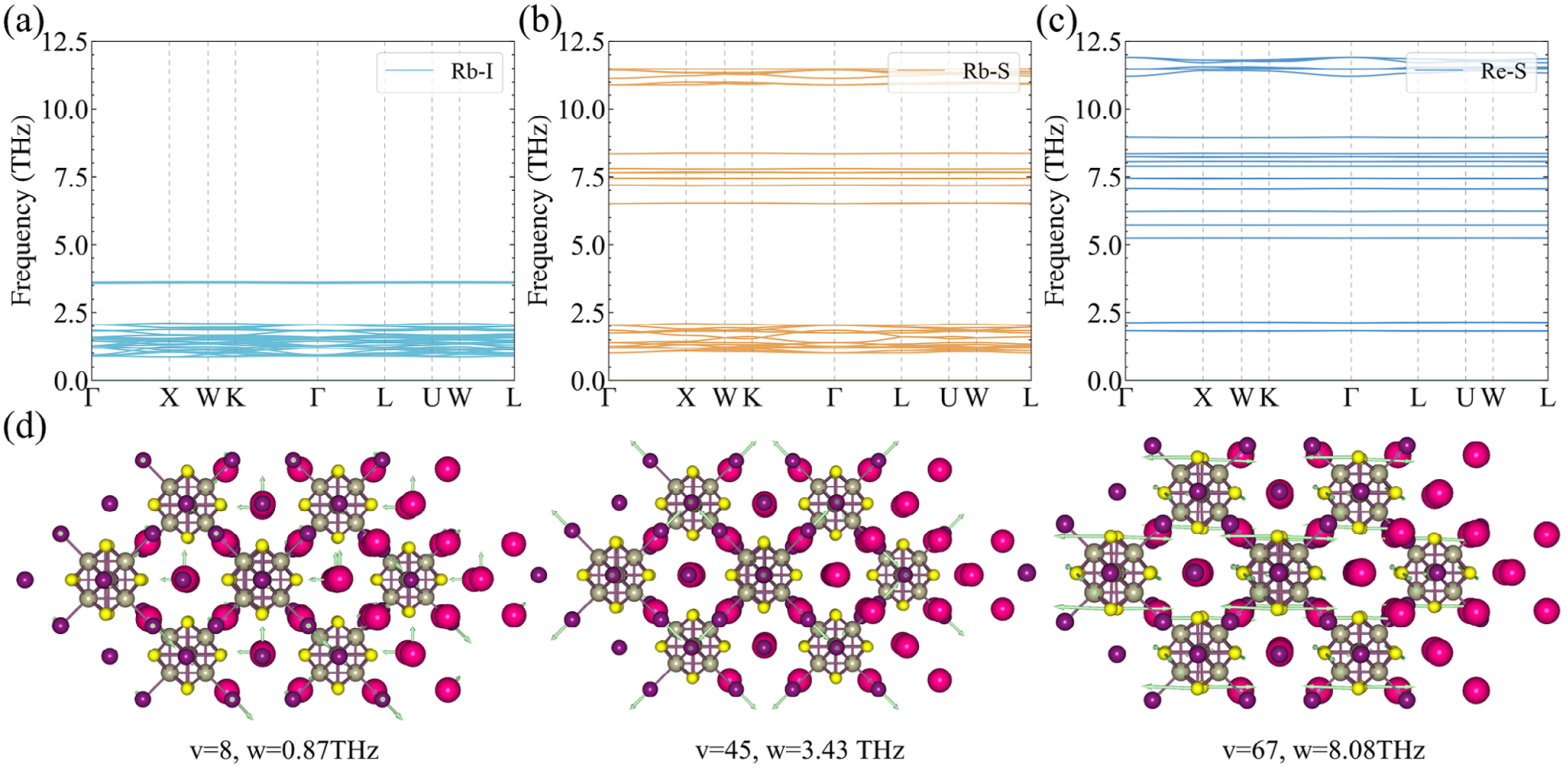
a) The phonon dispersion between Rb and I, b) between Rb and S, and c)between Re and S for Rb_6_Re_6_S_8_I_8_ at 300 K. The visualization of phonon mode at point in e) low‐frequency ω = 0.87 THz, f) low‐medium‐frequency ω = 3.47 THz, and g) high‐frequency ω = 8.08 THz optical branches.v is the v‐th phonon branch.

The strength of interatomic interactions can be characterized through force constants. Figure 2d displays the second‐order average interaction force constants (|Φ_2_|) between key atomic pairs. Both Re–S and Re–I1 pairs exhibit large |Φ_2_|, confirming the formation of stable [Re_6_S_8_I_6_]^4 −^ octahedral clusters. In contrast, the weak interactions between alkali metal cations X^+^ and ionic I^−^ suggest that X_6_Re_6_S_8_I_8_ can be viewed as mobile X^+^ cations and ionic $I_{2}^{-}$ species within the rigid [Re_6_S_8_I_6_]^4 −^ framework. To explicitly demonstrate the origin of phonon band flattening, we reconstructed phonon spectra by selectively retaining specific interatomic force constants, as shown in **Figure** **3**a–c. Notably, all retained interactions produce exceptionally flat phonon dispersions except for Re–S‐derived optical branches above 10 THz, where stronger bonding induces greater dispersion. Figure 3d illustrates characteristic optical phonon modes across frequency domains at Γ point. The low‐frequency modes primarily correspond to the relative vibrations between Rb and I, the mid‐frequency modes are attributed to the vibrations of I relative to the [Re_6_S_8_I_6_]^4 −^ cluster, and the high‐frequency modes arise from the internal atomic vibrations within the [Re_6_S_8_I_6_]^4 −^ cluster. This mode localization hierarchy directly correlates with the hierarchy of bonding strengths revealed by |Φ_2_| analysis.

**Figure 4:**
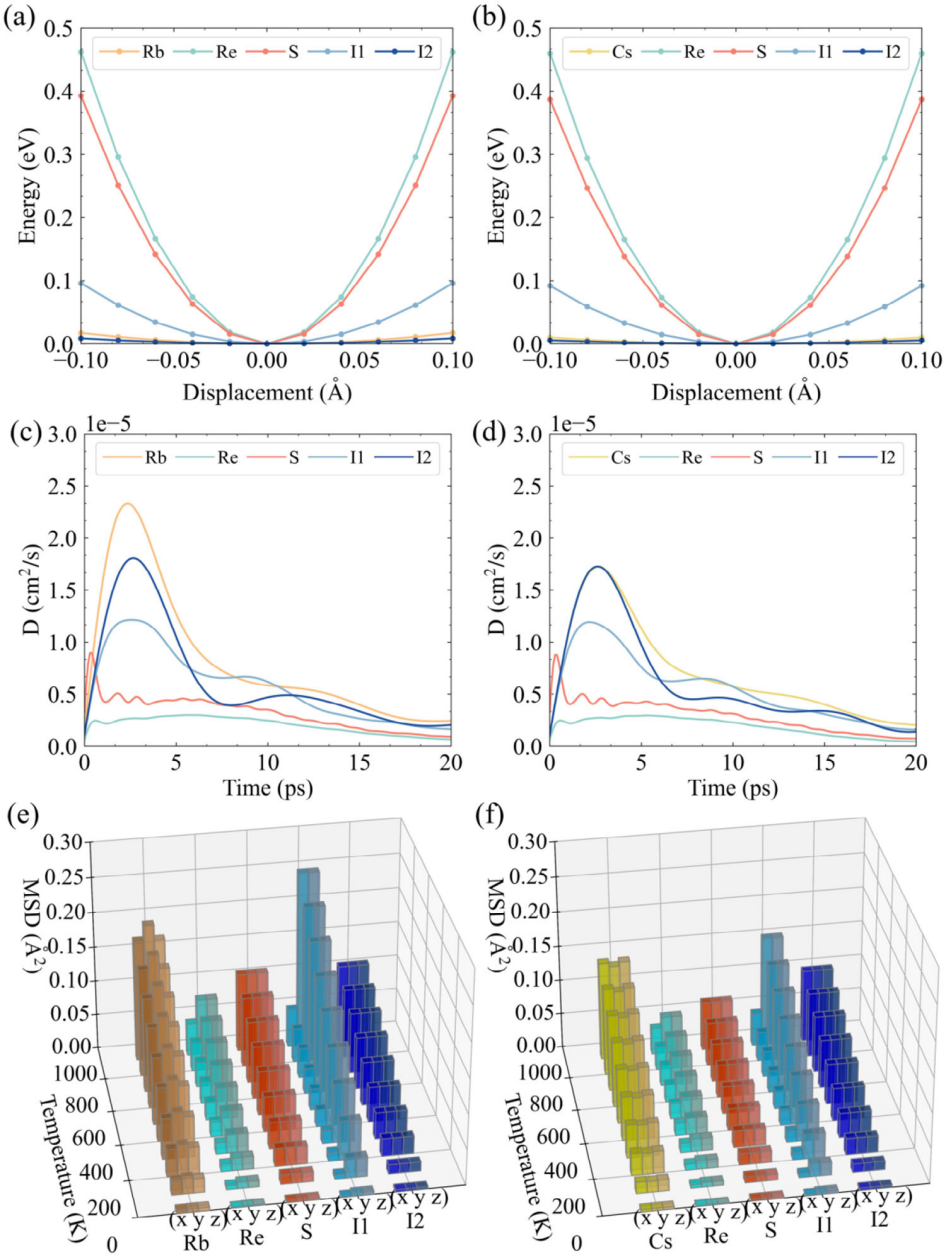
The potential energy as a function of the atomic displacement of a) Rb_6_Re_6_S_8_I_8_, and b) Cs_6_Re_6_S_8_I_8_ along the a‐direction. The diffusion coefficient of c) Rb_6_Re_6_S_8_I_8_, and d) Cs_6_Re_6_S_8_I_8_ d) at 300 K. The temperature‐dependent MSD of e) Rb_6_Re_6_S_8_I_8_, and f) Cs_6_Re_6_S_8_I_8_.

**Figure** **4**a,b presents the potential energy curves for atomic displacements along the *a*‐axis in X_6_Re_6_S_8_I_8_ (X = Rb, Cs). The steepest potential energy curves correspond to Re and S atoms, indicating strong resistance to displacement from their local environments. In contrast, remarkably flat potential energy curves are observed for X and I2 atoms, suggesting their high mobility within the lattice. The overall lattice anharmonicity primarily stems from the motion of X and I atoms. Notably, the potential energy landscapes surrounding each atom type in Rb_6_Re_6_S_8_I_8_ and Cs_6_Re_6_S_8_I_8_ exhibit nearly identical chemical environments. Figure 4c,d display the diffusion coefficients at 300 K, revealing that X and I atoms exhibit diffusion coefficients (*D*) more than twice as large as those of Re and S atoms. This further confirms the loosely bound nature of Rb and I atoms moving between [Re_6_S_8_I_6_]^4−^ clusters, while Re and S atoms remain constrained within the clusters. The temperature‐dependent mean square displacements (MSDs) are shown in Figure 4e,f. All atoms demonstrate increased MSDs at higher temperatures, with X and I2 atoms showing particularly large displacements in *x*, *y*, and *z* directions. Interestingly, I1 exhibits anisotropic behavior ‐ small MSD along *x*‐direction but large along *y* and *z*‐directions, reflecting distinct chemical environments. Compared with Cs_6_Re_6_S_8_I_8_, the Rb_6_Re_6_S_8_I_8_ compound demonstrates greater temperature‐induced variations in MSDs, consistent with its softer lattice dynamics as evidenced by sound velocity and |Φ_2_| analysis.

**Figure 5:**
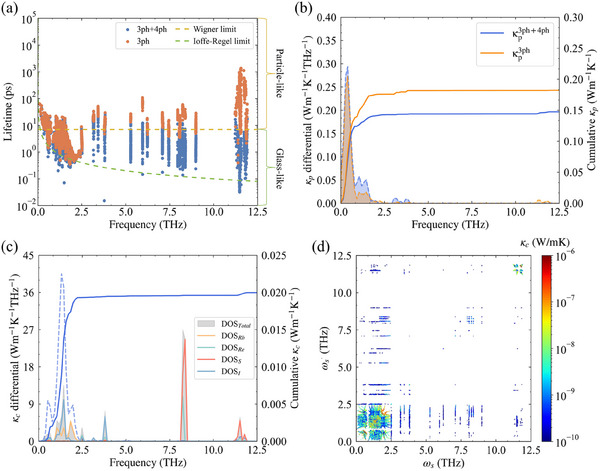
a) The phonon lifetimes of the 3ph and 3ph+4ph as a function of phonon frequencies for Rb_6_Re_6_S_8_I_8_ at 300 K. Where the Ioffe‐Regel ($\tau =\frac{1}{\omega }$) and Wigner ($\tau =\frac{1}{\Delta \omega_{\mathrm{avg}}}$) limits are represented by dotted yellow lines and green lines. b) Calculated cumulative and differential $\kappa_{p}$ as a function of phonon frequencies for Rb_6_Re_6_S_8_I_8_ at 300 K. c) Calculated cumulative and differential $\kappa_{c}$ as a function of phonon frequencies for Rb_6_Re_6_S_8_I_8_ at 300 K. d) The resolved $\kappa_{c}$ associated with various pairs of phonon frequencies ($\omega_{s}$ and $\omega_{s^{\prime }}$).

As shown in **Figure** **5**b and Figure S7b (Supporting Information), the calculated differential and cumulative $\kappa_{p}$ of X_6_Re_6_S_8_I_8_ (X = Rb, Cs) at 300 K is derived using temperature‐dependent force constants. The particle‐like thermal conductivity component $\kappa_{p}^{3\mathrm{ph}}$ for Rb_6_Re_6_S_8_I_8_ and Cs_6_Re_6_S_8_I_8_ under pure three‐phonon (3ph) scattering are 0.17 and 0.18 W m^−1^ K^−1^, respectively. Upon inclusion of four‐phonon (4ph) scattering, the $\kappa_{p}^{3\mathrm{ph}+4\mathrm{ph}}$ values decrease to 0.15 and 0.17  W m^−1^ K^−1^, with the Rb‐analogue exhibiting a reduction exceeding 10%, indicating the non‐negligible contribution of four‐phonon scattering. More intuitively, Figure S8 (Supporting Information) presents the obtained $\kappa_{p}$ and $\kappa_{c}$ under 3ph and 3ph+4ph scattering mechanisms. The $\kappa_{p}^{3\mathrm{ph}+4\mathrm{ph}}$ values of X_6_Re_6_S_8_I_8_ are significantly lower than those of other superionic crystals, e.g., the reported $\kappa_{p}^{3\mathrm{ph}+4\mathrm{ph}}$ is 0.32 W m^−1^ K^−1^ for Re_6_Se_8_Cl_2_.^[^ ^36^ ^]^ This reduction can be attributed to the structural complexity of the primitive cell. Specifically, Rb_6_Re_6_S_8_I_8_ exhibits a more complex primitive cell containing 28 atoms,^[^ ^37^ ^]^ compared to only 16 atoms in Re_6_Se_8_Cl_2_. The κ_p_ generally decreases with increasing number of atoms per primitive cell, as exemplified by Ag_9_GaS_6_ containing 56 atoms per primitive cell, which exhibits an ultralow κ_p_ = 0.031 W m^−1^ K^−1^.^[^ ^38^ ^]^ This negative correlation arises from enhanced phonon scattering channels in systems with larger primitive cells.

Figure 5b reveals two distinct growth regions of $\kappa_{p}^{3\mathrm{ph}+4\mathrm{ph}}$ in Rb_6_Re_6_S_8_I_8_: a low‐frequency regime (0–0.8 THz) contributing 83% of the total thermal conductivity, dominated by acoustic branches and the three lowest‐frequency optical branches, followed by a low‐frequency regime (0.8–1.7 THz) contributing 11% from low‐lying optical modes. A similar frequency dependence is observed in Cs_6_Re_6_S_8_I_8_, where phonons below 2.5 THz account for 95% of $\kappa_{p}^{3\mathrm{ph}+4\mathrm{ph}}$. The phonon lifetime (τ) distributions at 300 K, presented in Figure 5a and Figure S7a (Supporting Information), demonstrate significant four‐phonon scattering effects. Most phonons exhibit lifetimes below 10 ps, coupled with low group velocities, collectively suppressing κ_p_. In contrast, low‐frequency phonons maintain extended lifetimes (>10 ps) and higher group velocities, leading to their dominant contributions to κ_p_.

Within the framework of the unified lattice thermal transport theory,^[^ ^12^, ^13^ ^]^ the structural complexity of crystals inherently generates substantial off‐diagonal thermal conductivity components κ_c_ arising from wave‐like phonon tunneling. As shown in Figure 5a and Figure S7a (Supporting Information), phonon modes with lifetimes exceeding the Wigner limit ($\tau >\Delta \omega_{avg}^{-1}$) predominantly contribute to the particle‐like thermal conductivity κ_p_, while those with lifetimes between the Wigner and Ioffe‐Regel limits ($\omega^{-1}<\tau <\Delta \omega_{avg}^{-1}$) primarily govern the wave‐like tunneling component κ_c_. Here, $\Delta \omega_{\mathrm{avg}}$ is the average phonon band spacing, defined as $\Delta \omega_{\mathrm{avg}}=\frac{\omega_{\max}}{3N}$, $\omega_{\max}$ is the highest phonon frequency, and N is the number of atoms in the primitive cell. In the inclusion of 4ph scattering significantly reduces phonon lifetimes in Rb_6_Re_6_S_8_I_8_, driving numerous optical branches into the wave‐like tunneling regime, which results in a pronounced reduction in $\kappa_{p}^{3\mathrm{ph}+4\mathrm{ph}}$. Despite the abundance of phonon modes in the wave‐like tunneling regime, their contributions to κ_c_ remain negligible due to the predominance of mid‐ to high‐frequency optical modes characterized by flat phonon dispersion and large frequency gaps. This is quantitatively demonstrated in Figure 5c and Figure S7c, which present the frequency‐resolved cumulative κ_c_ spectrum, its differential contribution, and atom‐projected phonon density of states (DOS) at 300 K. Both Rb_6_Re_6_S_8_I_8_ and Cs_6_Re_6_S_8_I_8_ exhibit remarkably small κ_c_ values of 0.02 and 0.018 W·m^−1^·K^−1^, respectively, constituting only 12% and 10% of their total lattice thermal conductivity. The primary κ_c_ contributions in Rb_6_Re_6_S_8_I_8_ originate from phonon modes below 2.5 THz, as evidenced by three distinct spectral peaks. To elucidate their origins, Figure 5d maps the pairwise frequency combinations (ω_*s*_, $\omega_{s^{\prime }}$) contributing to κ_c_. The three peaks respectively arise from interactions between: i) acoustic and the lowest optical branches, ii) acoustic and low‐lying optical branches, and iii) low‐lying optical branches themselves. DOS analysis reveals that these spectral features predominantly stem from vibrations involving Rb and I atoms. Notably, high‐frequency optical modes above 11 THz exhibit non‐negligible κ_c_ contributions due to their dispersive phonon band structures and strong mode hybridization. While Cs_6_Re_6_S_8_I_8_ shows similar κ_c_ characteristics, so it will not be discussed repeatedly.

**Figure 6:**
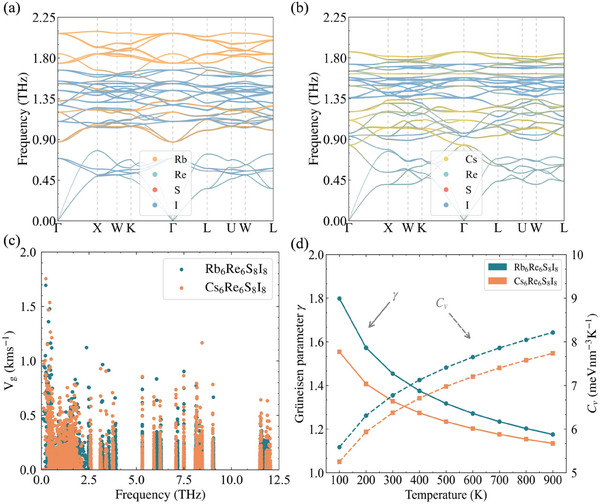
The low‐frequency atomic projected phonon dispersion of a) Rb_6_Re_6_S_8_I_8_, and b) Cs_6_Re_6_S_8_I_8_ at 300 K. c) The phonon group velocities as functions of thephonon frequency for X_6_Re_6_S_8_I_8_ (X = Rb, Cs) at 300 K. d) The gruneisen parameter (|γ|) and the heat capacity (C_*v*_) for X_6_Re_6_S_8_I_8_ (X = Rb, Cs) at 300 K.

A notable thermal transport anomaly emerges in X_6_Re_6_S_8_I_8_: Despite heavier atomic mass, Cs_6_Re_6_S_8_I_8_ exhibits larger lattice thermal conductivity than Rb_6_Re_6_S_8_I_8_. The lattice thermal conductivities obtained by replacing the mass of Rb in Rb_6_Re_6_S_8_I_8_ with those of Na, K, and Cs are shown in Table SII (Supporting Information). It can be seen that, without changing the force constants, the lattice thermal conductivity decreases with increasing mass. Therefore, the lower lattice thermal conductivity of Rb_6_Re_6_S_8_I_8_ compared to Cs_6_Re_6_S_8_I_8_ is not caused by mass but rather by the force constants. In addition, when substituting Rb with Cs, the $\kappa_{L}$ shows minimal change, whereas replacement with Na leads to significant variation. This occurs because Rb→Cs substitution induces negligible modification in the phonon spectrum, while Rb→Na substitution markedly enhances the dispersion of optical branches between 1.8–4.5 THz, as shown in Figure S9 (Supporting Information). Figure 2d reveals minimal changes in Φ_2_(Re‐S) and Φ_2_(Re‐I1) from Rb_6_Re_6_S_8_I_8_ to Cs_6_Re_6_S_8_I_8_, while Φ_2_(Re‐I2) decreases by 9.7%. In contrast, Φ_2_(Cs‐I1) and Φ_2_(Cs‐I2) increase by 16.5% and 23.6% compared to Φ_2_(Rb‐I1) and Φ_2_(Rb‐I2), respectively. As Re‐I1 interactions mainly affect mid‐frequency optical branches with negligible $\kappa_{L}$ contribution, the anomaly originates from enhanced X‐I interactions. Bader charge analysis confirms stronger ionic bonding in Cs_6_Re_6_S_8_I_8_, with greater charge transfer between Cs and I2. X‐I interactions predominantly modify acoustic and low‐frequency optical branches (**Figure** **6**a,b). Rb_6_Re_6_S_8_I_8_ displays a small bandgap near 0.8 THz, flattening the lowest three optical branches and reducing phonon group velocities below 1 THz (Figure 6c). Substituting Φ_2_(Cs‐I) in Cs_6_Re_6_S_8_I_8_ with Φ_2_(Rb‐I) softens phonon spectra (Figure S10, Supporting Information). Weaker Rb‐I interactions enhance anharmonicity, evidenced by higher *C*_*v*_ and |γ| values in Rb_6_Re_6_S_8_I_8_ compared to Cs_6_Re_6_S_8_I_8_ at identical temperatures (Figure 6d).

**Figure 7:**
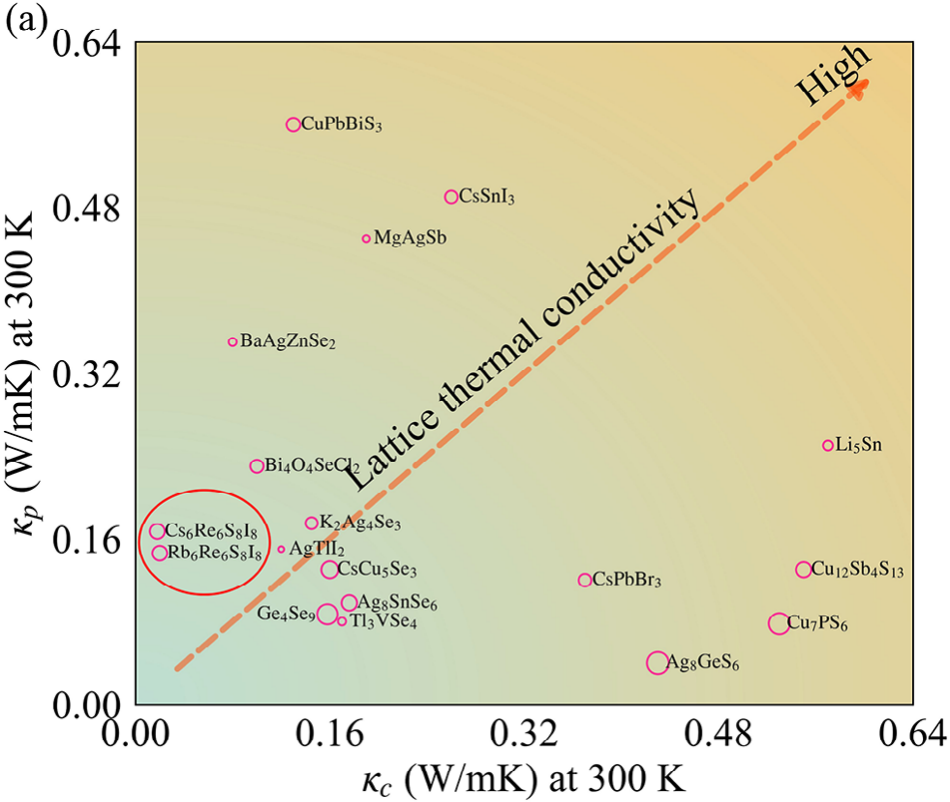
Potential pathways for pushing κ to its lower limit in inorganic materials. The dashed red line represents the current protocol to find materials with lower thermal conductivity, and the size of the pink circles is proportional to the number of atoms within the primitive cell. Lattice thermal conductivities are extracted from previous studies based on unified theory.^[^ ^11^, ^12^, ^40^, ^41^, ^42^, ^43^, ^44^, ^45^, ^46^, ^47^, ^48^, ^49^, ^50^, ^51^, ^52^ ^]^ For anisotropic materials, the average values are used.

The coexistence of ultralow particle‐like κ_p_ and wave‐like κ_c_ in X_6_Re_6_S_8_I_8_ represents a rare phenomenon in crystalline materials. While complex crystal structures generally exhibit suppressed κ_p_ through enhanced phonon scattering (due to increased number of atoms in PC and Brillouin zone folding effects), excessive structural complexity may paradoxically enhance κ_c_. For instance, skutterudite YbFe_4_Sb_12_ (17 atoms in PC)^[^ ^39^ ^]^ shows κ_p_ = 0.83 W m^−1^ K^−1^, and Bi_4_O_4_SeCl_2_ (22 atoms in PC)^[^ ^40^ ^]^ achieves κ_p_ = 0.23 W m^−1^ K^−1^. However, further increasing crystal complexity (e.g., Cu_7_PS_6_ with 56 atoms/primitive cell^[^ ^38^ ^]^) reduces the average phonon band spacing Δω_avg_, leading to non‐negligible κ_c_ contributions exceeding 87%. **Figure** **7** maps the room‐temperature κ_p_ and κ_c_ values against primitive cell atom counts for various materials. As shown in Figure 7, the X_6_Re_6_S_8_I_8_ system has a moderately complex lattice (with 28 atoms in PC), and its $\kappa_{p}$ is not the lowest. However, it exhibits both low $\kappa_{p}$ and extremely low $\kappa_{c}$, resulting in the lowest total κ_L_. In the X_6_Re_6_S_8_I_8_ system, the large difference in bond strength within and between [Re_6_S_8_I_6_]^4 −^ clusters promotes phonon localization, generating extensive discrete phonon flat bands. These phonon flat bands reduce $\kappa_{c}$ in two ways: first, through low phonon group velocities, and second, through large interband gaps. These characteristics contrast with systems like AgTlI_2_^[^ ^11^ ^]^ where balanced κ_p_/κ_c_ contributions (≈50% each) yield κ_L_ = 0.25 W·m^−1^·K^−1^, demonstrating the critical role of selective phonon engineering in achieving ultralow thermal conductivity.

## 4 Conclusion

In conclusion, based on the unified theory of lattice thermal transport, we have demonstrated that the superionic crystals Rb_6_Re_6_S_8_I_8_ and Cs_6_Re_6_S_8_I_8_ achieve ultralow lattice thermal conductivity κ_L_ of 0.17 and 0.19 W m^−1^ K^−1^ at 300 K, respectively, with weak temperature dependence. This exceptional behavior stems from their unique hierarchical architecture: strongly bonded [Re_6_S_8_I_6_]^4 −^ clusters interconnected via weak ionic interactions between X^+^ (X = Rb, Cs) and I^−^ ions. The relative vibrations between clusters generate extensive optical phonon flat bands, suppressing coherent thermal transport (κ_c_ ≈ 0.02 W m^−1^ K^−1^). Concurrently, the soft X^+^‐I^−^ interactions depress both low‐frequency acoustic branches and optical modes, resulting in diminished particle‐like conductivity (κ_p_ = 0.15 and 0.17 W m^−1^ K^−1^). Notably, Rb_6_Re_6_S_8_I_8_ exhibits anomalous thermal transport characteristics despite its lower atomic mass compared to the Cs analogue. The weaker Rb^+^‐I^−^ coupling enhances lattice anharmonicity, reduces average phonon group velocities, and introduces a phonon bandgap that further restricts dispersion in low‐lying optical branches. These synergistic effects collectively yield the record‐low κ_L_ in Rb_6_Re_6_S_8_I_8_. Our findings establish a new paradigm for phonon engineering through cluster‐based anharmonicity tuning, providing a roadmap to develop ultralow κ_L_ materials for thermoelectric and thermal barrier applications.

## Conflict of Interest

The authors declare no conflict of interest.

## Supporting information

Supporting Information

## Data Availability

The data that support the findings of this study are available from the corresponding author upon reasonable request.
