# Supplementary material for: Decoupling Ultralow Coherent and Particle‐Like Phonon Transport via Bonding Hierarchy in Soft Superionic Crystals
Source: Adv Sci (Weinh). 2025 Jun 5;12(31):e06807. doi: 10.1002/advs.202506807 (PMC12376585; doi:10.1002/advs.202506807)
Supplement: Supplementary file 1 — Supporting Information [file ADVS-12-e06807-s001.pdf]

ADVANCED SCIENCE
Open Access

Supporting Information

for *Adv. Sci.*, DOI 10.1002/advs.202506807

Decoupling Ultralow Coherent and Particle-Like Phonon Transport via Bonding Hierarchy in Soft Superionic Crystals

Wenjie Xiong, Hao Huang, Yu Wu, Xinji Xu, Geng Li, Zonglin Gu* and Shuming Zeng**

Support Information for 'Decoupling Ultralow Coherent and Particle-Like Phonon Transport via Bonding Hierarchy in Soft Superionic Crystals'

Wenjie Xiong,[1] Hao Huang,[2] Yu Wu,[3,*] Xinji Xu,[1]
Geng Li,[4,5] Zonglin Gu,[1,†] and Shuming Zeng[1,‡]

[1]*College of Physics Science and Technology, Yangzhou University, Jiangsu, 225009, China*

[2]*Advanced Copper Industry College, Jiangxi University of Science and Technology, Yingtan, 335000,China*

[3]*Advanced Thermal Management Technology and Functional Materials Laboratory, Ministry of Education Key Laboratory of NSLSCS, School of Energy and Mechanical Engineering, Nanjing Normal University, Jiangsu, Nanjing, 210023, China*

[4]*China Rare Earth Group Research Institute, Shenzhen, Guangdong, 518000, China*

[5]*Key Laboratory of Rare Earths, Ganjiang Innovation Academy, Chinese Academy of Sciences, Ganzhou, 341000, China*

(Dated: May 11, 2025)

* wuyu@njnu.edu.cn
† guzonglin@yzu.edu.cn
‡ zengsm@yzu.edu.cn

TABLE SI. Crystal structure parameters, atomic distances, electronic properties of $X_6Re_6S_8I_8$ (X = Rb, Cs)

Compounds	Lattice Constants						Atomic Distances (Å)					E_g (eV)	E_{Fermi} (eV)
	a	b	c	α (°)	β (°)	γ (°)	X-I1	X-I2	Re-Re	Re-S	Re-I1		
$Rb_6Re_6S_8I_8$	10.961	10.961	10.961	60	60	60	3.942	3.875	2.601	2.405	2.757	2.422	1.771
$Cs_6Re_6S_8I_8$	11.182	11.182	11.182	60	60	60	4.007	3.953	2.605	2.404	2.764	2.454	1.834

TABLE SII. The lattice thermal conductivity obtained by replacing the mass of Rb in $Rb_6Re_6S_8I_8$ with the masses of Na, K, and Cs at 300 K, unit in $Wm^{-1}K^{-1}$.

	Rb→Na	Rb→K	Rb→Rb	Rb→Cs
κ_L	0.242	0.211	0.167	0.164
κ_p	0.219	0.190	0.147	0.147
κ_c	0.023	0.021	0.020	0.017

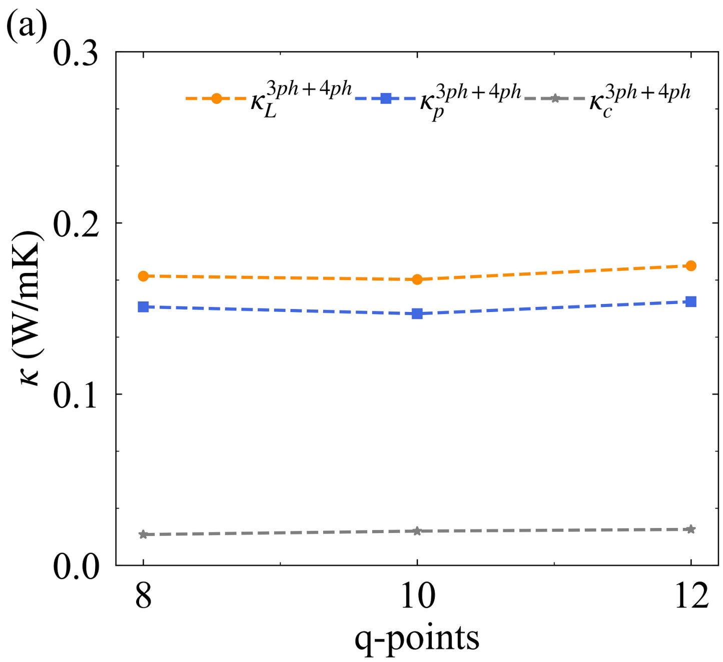

FIG. S1. The lattice thermal conductivities of $Rb_6Re_6S_8I_8$ calculated with different q-points ($8\times8\times8$, $10\times10\times10$, and $12\times12\times12$) at 300 K.

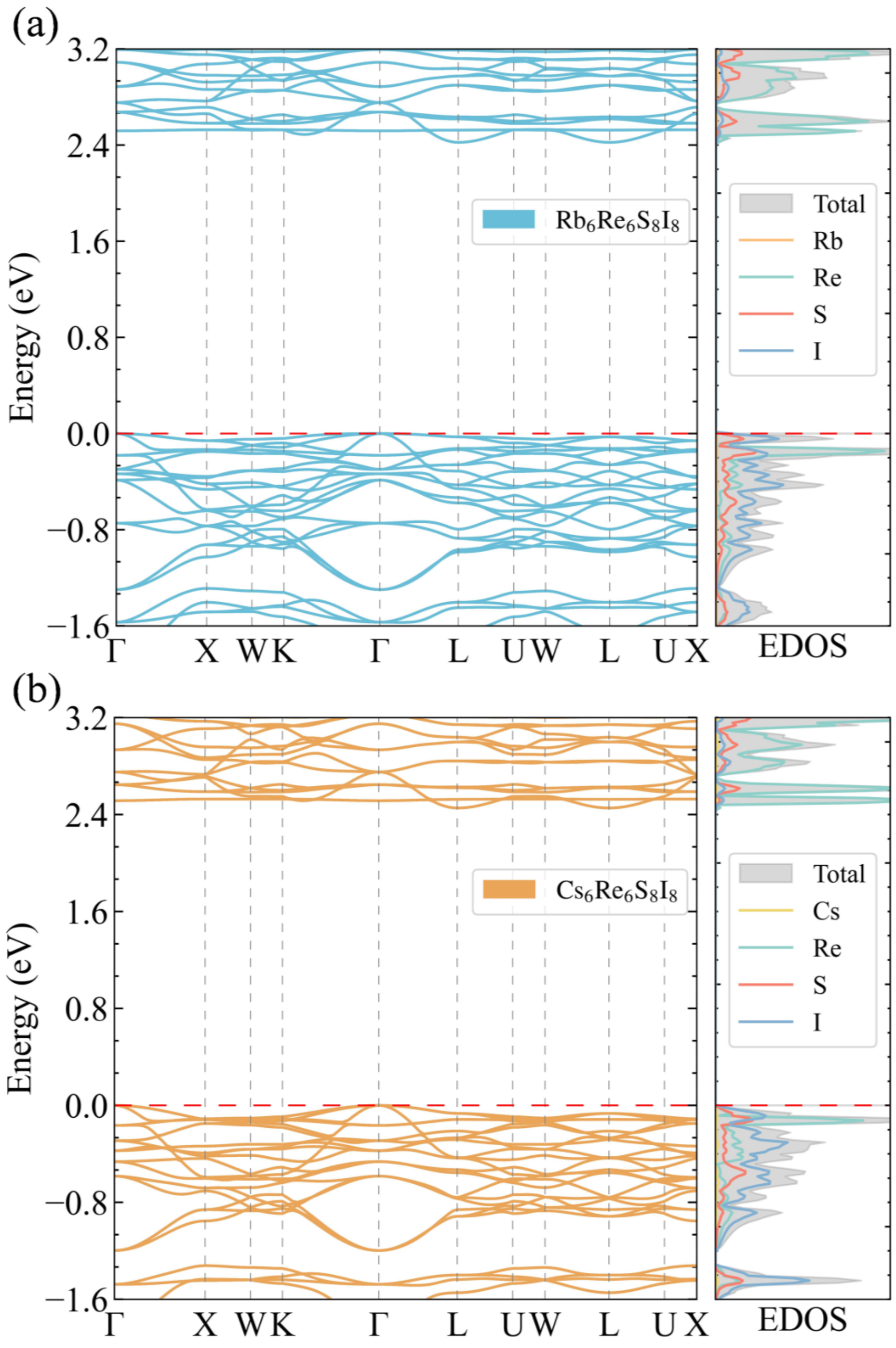

FIG. S2. The electronic structure and partial electronic density of states for (a) $Rb_6Re_6S_8I_8$ and (b) $Cs_6Re_6S_8I_8$.

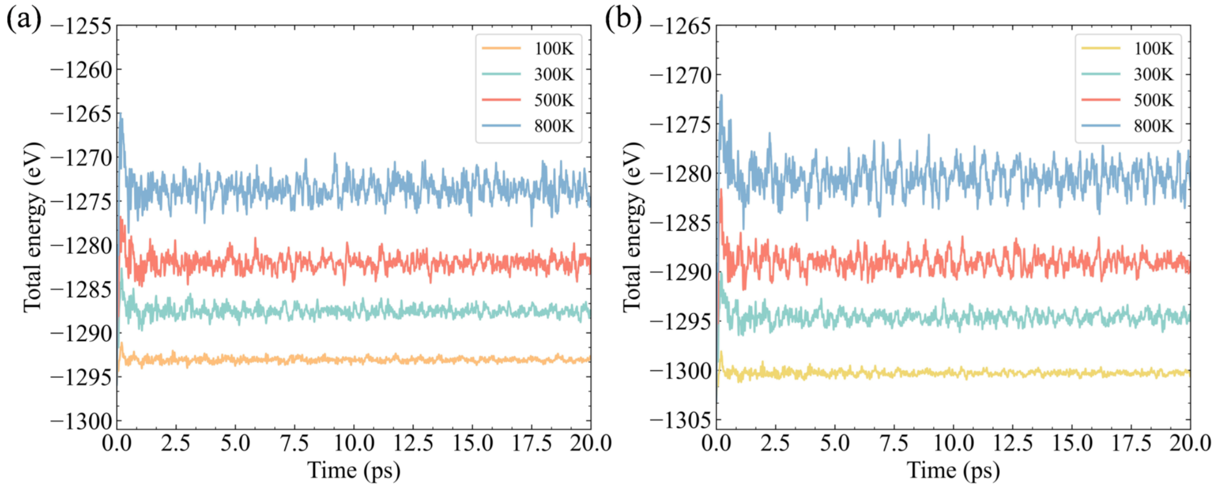

FIG. S3. The total energy of (a) $Rb_6Re_6S_8I_8$ and (b) $Cs_6Re_6S_8I_8$ at different temperatures as a function of time.

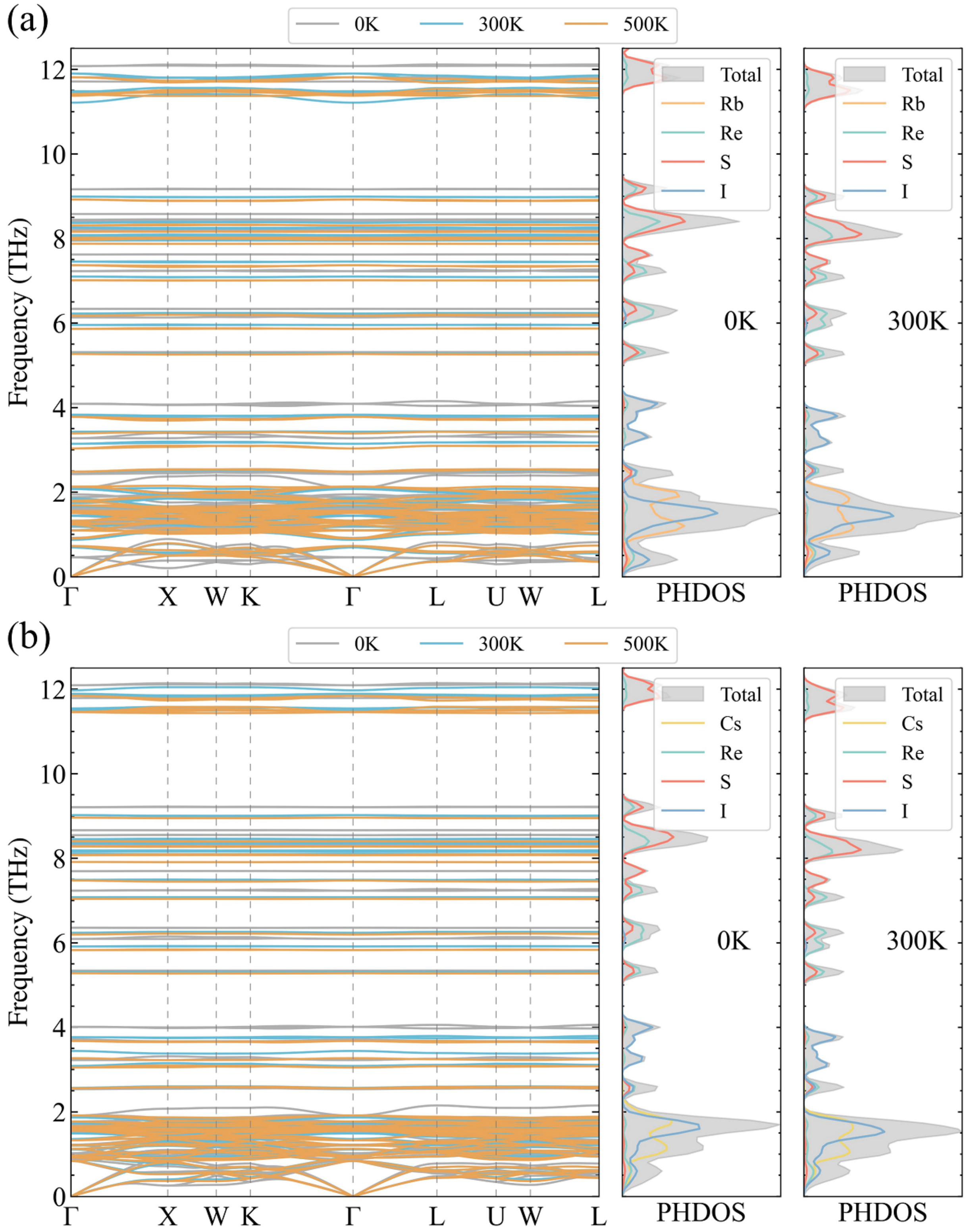

FIG. S4. a) The temperature-dependent phonon dispersion curves and associated phonon densities of states (PHDOS) for (a) $Rb_6Re_6S_8I_8$ and (b) $Cs_6Re_6S_8I_8$.

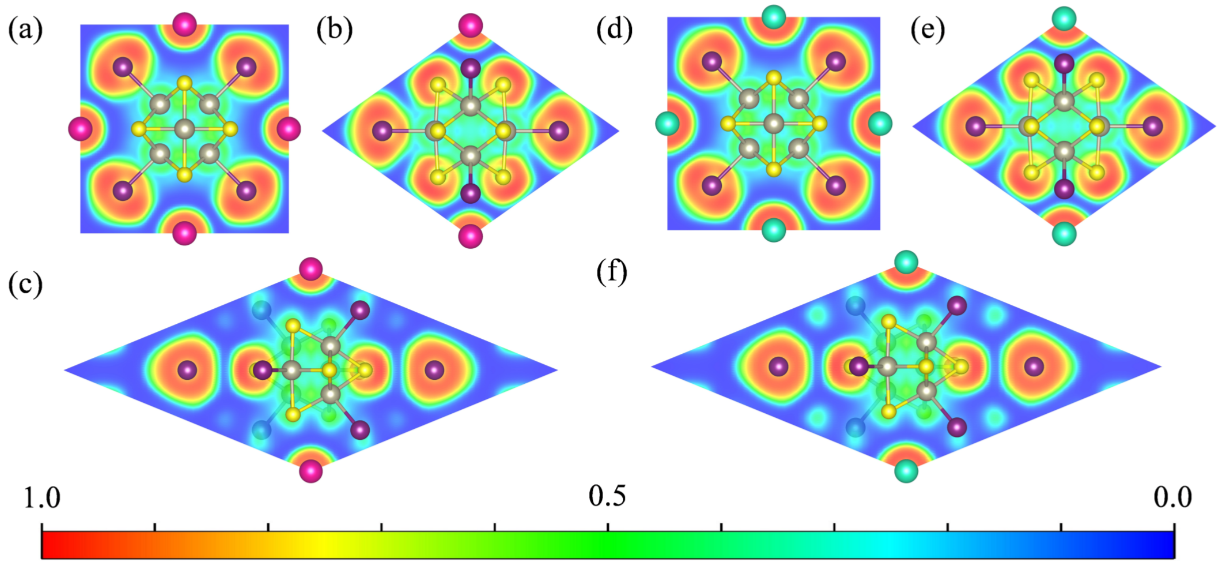

FIG. S5. The electronic localization function (ELF) corresponding to the (a) (0,1,1) plane, the (b) (1,2,1) plane, and the (c) (-1,2,-1) plane of $Rb_6Re_6S_8I_8$. The ELF corresponding to the (d) (0,1,1) plane, the (e) (1,2,1) plane, and the (f) (-1,2,-1) plane of $Cs_6Re_6S_8I_8$.

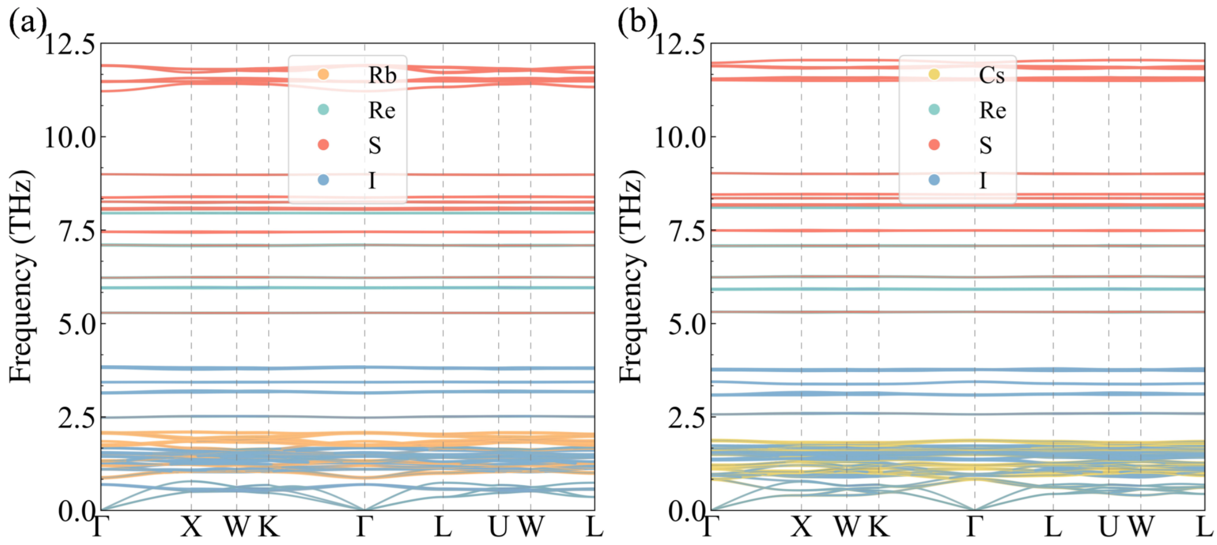

FIG. S6. The projected phonon dispersion of (a) $Rb_6Re_6S_8I_8$, and (b) $Cs_6Re_6S_8I_8$ weighted by their constituted atoms at 300 K.

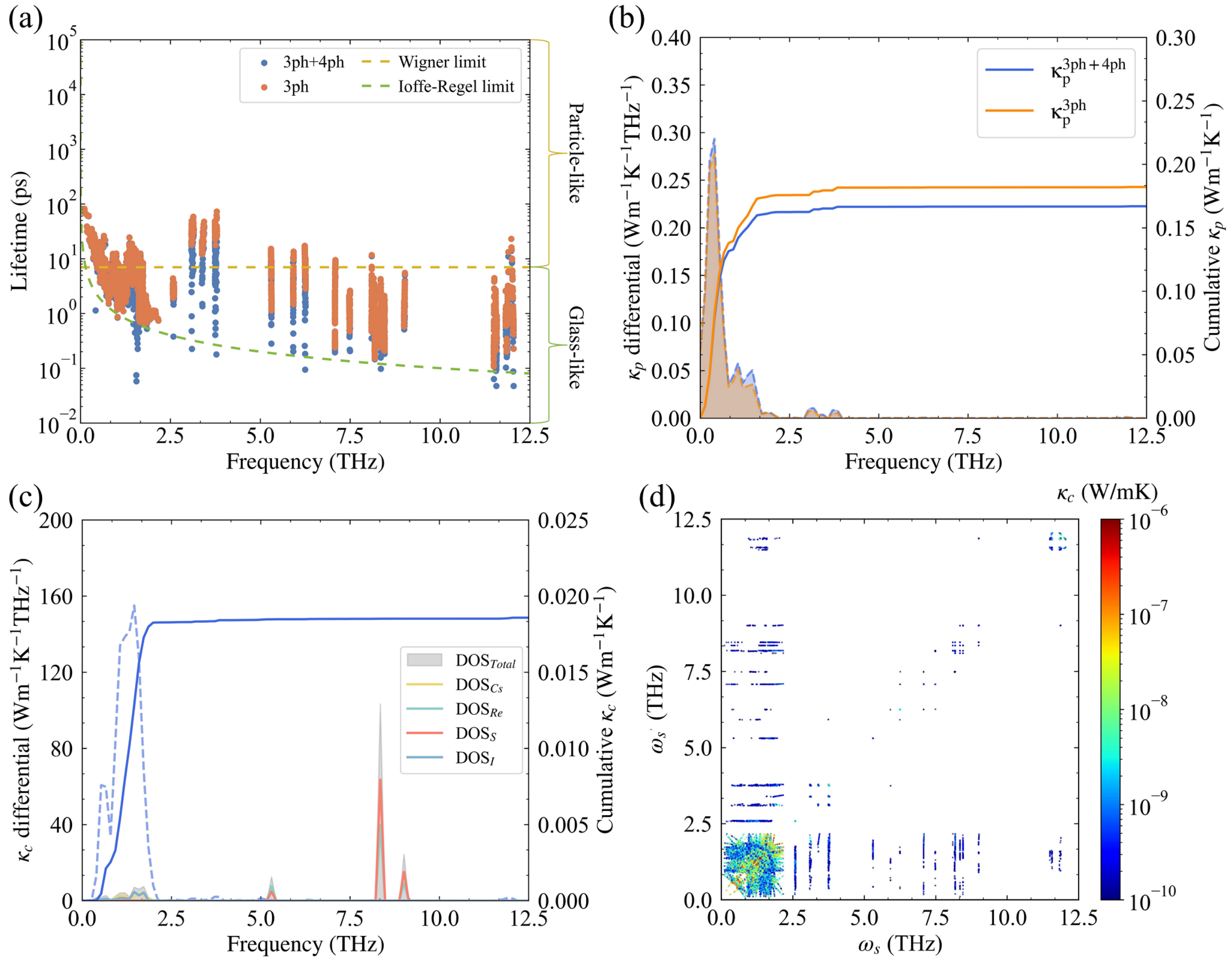

FIG. S7. (a) The phonon lifetimes of the 3ph and 3ph+4ph as a function of phonon frequencies for $Cs_6Re_6S_8I_8$ at 300 K. Where the Ioffe-Regel ($\tau = \frac{1}{\omega}$) and Wigner ($\tau = \frac{1}{\Delta\omega_{avg}}$) limits are represented by dotted yellow lines and green lines. (b) Calculated cumulative and differential κ_p as a function of phonon frequencies for $Rb_6Re_6S_8I_8$ at 300 K.(c) Calculated cumulative and differential κ_c as a function of phonon frequencies for $Cs_6Re_6S_8I_8$ at 300 K. (d) The resolved κ_c associated with various pairs of phonon frequencies (ω_s and $\omega_{s'}$).

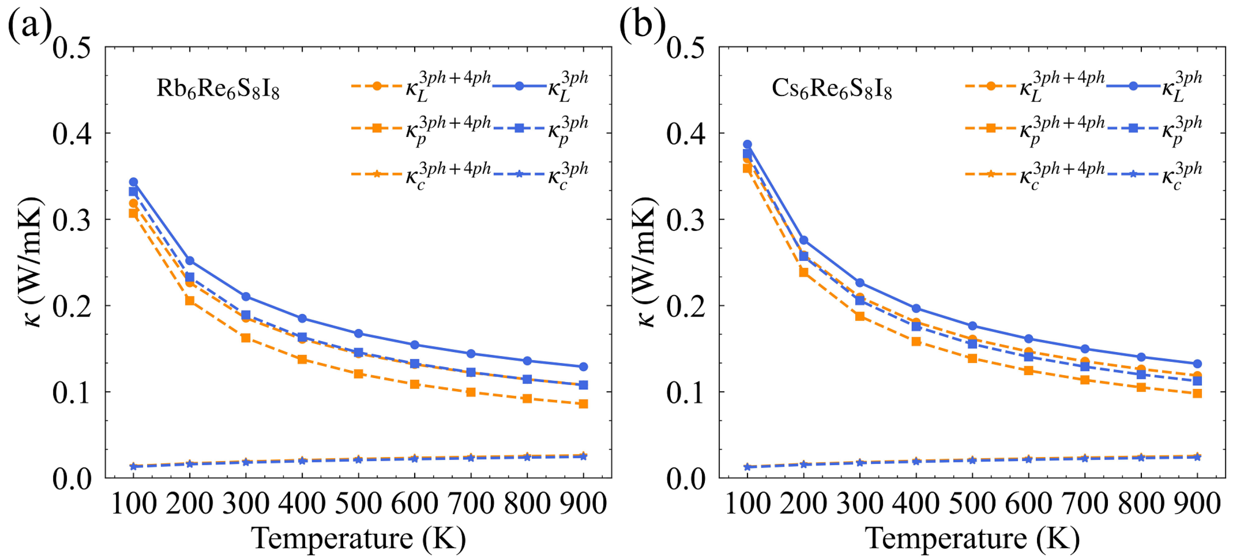

FIG. S8. The κ_{p} and κ_{c} of (a) $Rb_6Re_6S_8I_8$, and (b) $Cs_6Re_6S_8I_8$ calculated by including 3ph and 3ph+4ph scattering.

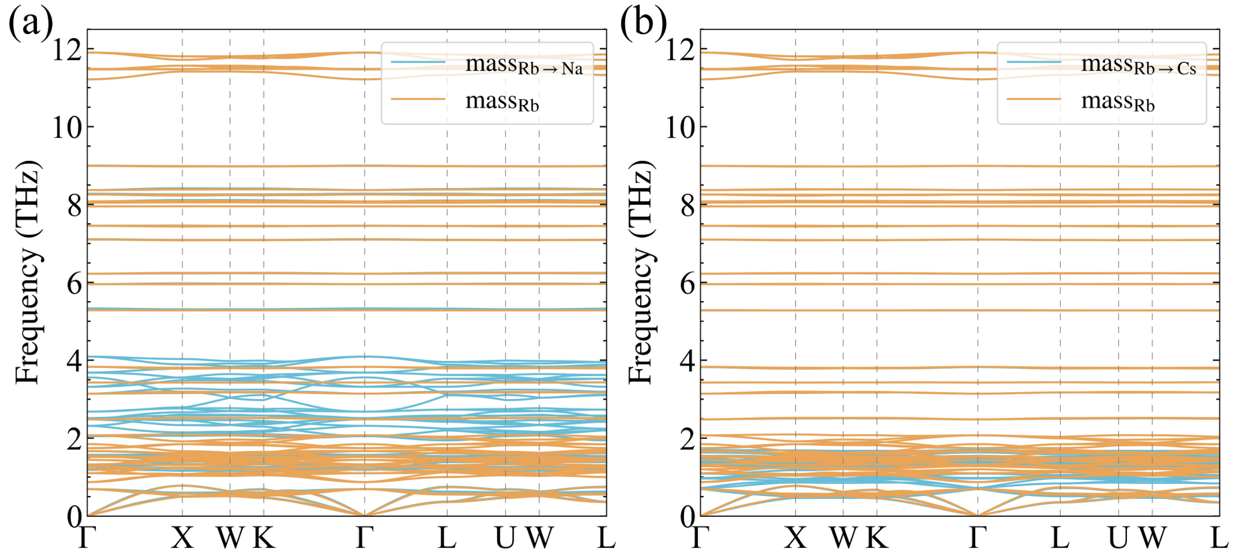

FIG. S9. The calculated phonon bands of $Rb_6Re_6S_8I_8$ with Rb replaced by (a) Na and (b) Cs at 300 K.

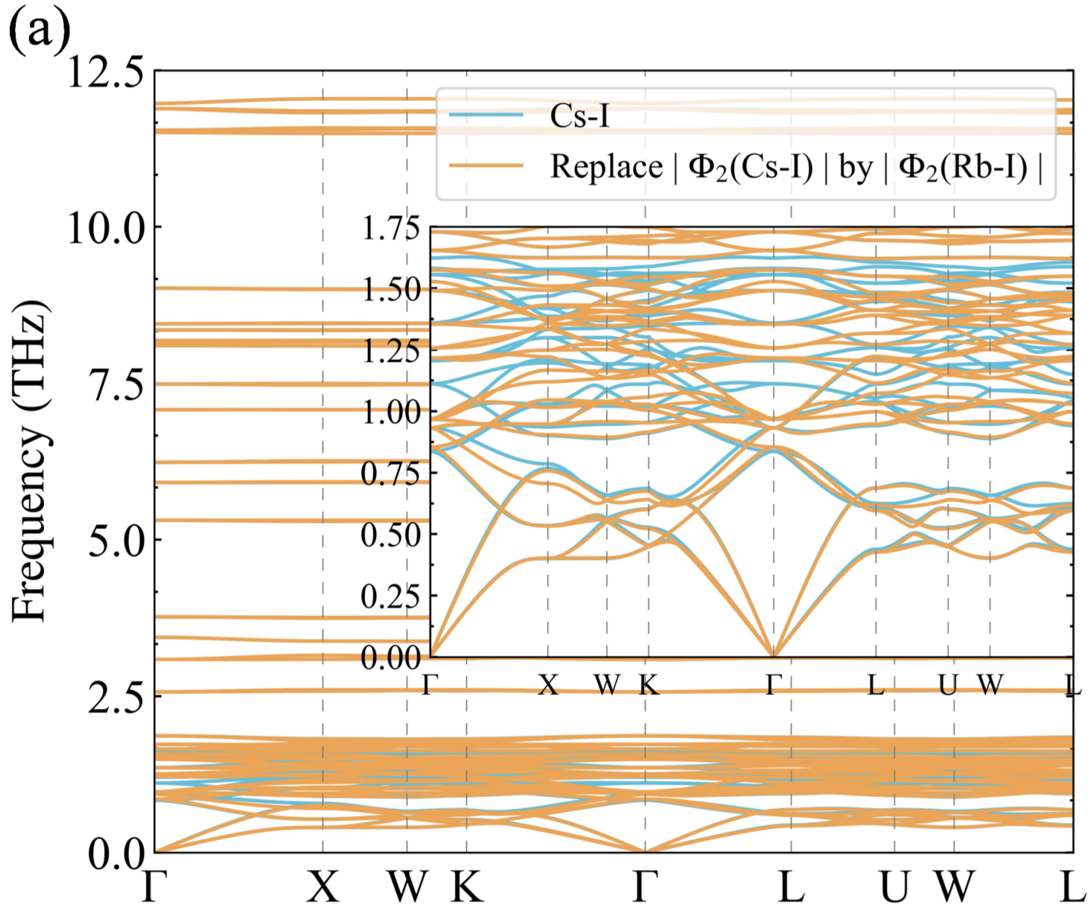

FIG. S10. a) The phonon dispersion of $Cs_6Re_6S_8I_8$ and that after replacing $|\Phi_2(Cs-I)|$ by $|\Phi_2(Rb-I)|$.
